# Systems metabolic engineering of *Corynebacterium glutamicum* for the production of the carbon-5 platform chemicals 5-aminovalerate and glutarate

**DOI:** 10.1186/s12934-016-0553-0

**Published:** 2016-09-13

**Authors:** Christina Maria Rohles, Gideon Gießelmann, Michael Kohlstedt, Christoph Wittmann, Judith Becker

**Affiliations:** Institute of Systems Biotechnology, Saarland University, Saarbrücken, Germany

**Keywords:** Bio-nylon, Synthetic biology, Metabolic engineering, Platform chemical, Lysine monooxygenase, Heterologous production, Bio-economy

## Abstract

**Background:**

The steadily growing world population and our ever luxurious life style, along with the simultaneously decreasing fossil resources has confronted modern society with the issue and need of finding renewable routes to accommodate for our demands. Shifting the production pipeline from raw oil to biomass requires efficient processes for numerous platform chemicals being produced with high yield, high titer and high productivity.

**Results:**

In the present work, we established a de novo bio-based production process for the two carbon-5 platform chemicals 5-aminovalerate and glutarate on basis of the lysine-hyperproducing strain *Corynebacterium glutamicum* LYS-12. Upon heterologous implementation of the *Pseudomonas putida* genes *davA*, encoding 5-aminovaleramidase and *davB*, encoding lysine monooxygenase, 5-aminovalerate production was established. Related to the presence of endogenous genes coding for 5-aminovalerate transaminase (*gabT*) and glutarate semialdehyde dehydrogenase, 5-aminovalerate was partially converted to glutarate. Moreover, residual l-lysine was secreted as by-product. The issue of by-product formation was then addressed by deletion of the *lysE* gene, encoding the l-lysine exporter. Additionally, a putative *gabT* gene was deleted to enhance 5-aminovalerate production. To fully exploit the performance of the optimized strain, fed-batch fermentation was carried out producing 28 g L^−1^ 5-aminovalerate with a maximal space–time yield of 0.9 g L^−1^ h^−1^.

**Conclusions:**

The present study describes the construction of a recombinant microbial cell factory for the production of carbon-5 platform chemicals. Beyond a basic proof-of-concept, we were able to specifically increase the production flux of 5-aminovalerate thereby generating a strain with excellent production performance. Additional improvement can be expected by removal of remaining by-product formation and bottlenecks, associated to the terminal pathway, to generate a strain being applicable as centerpiece for a bio-based production of 5-aminovalerate.

## Background

**Fig. 1:**
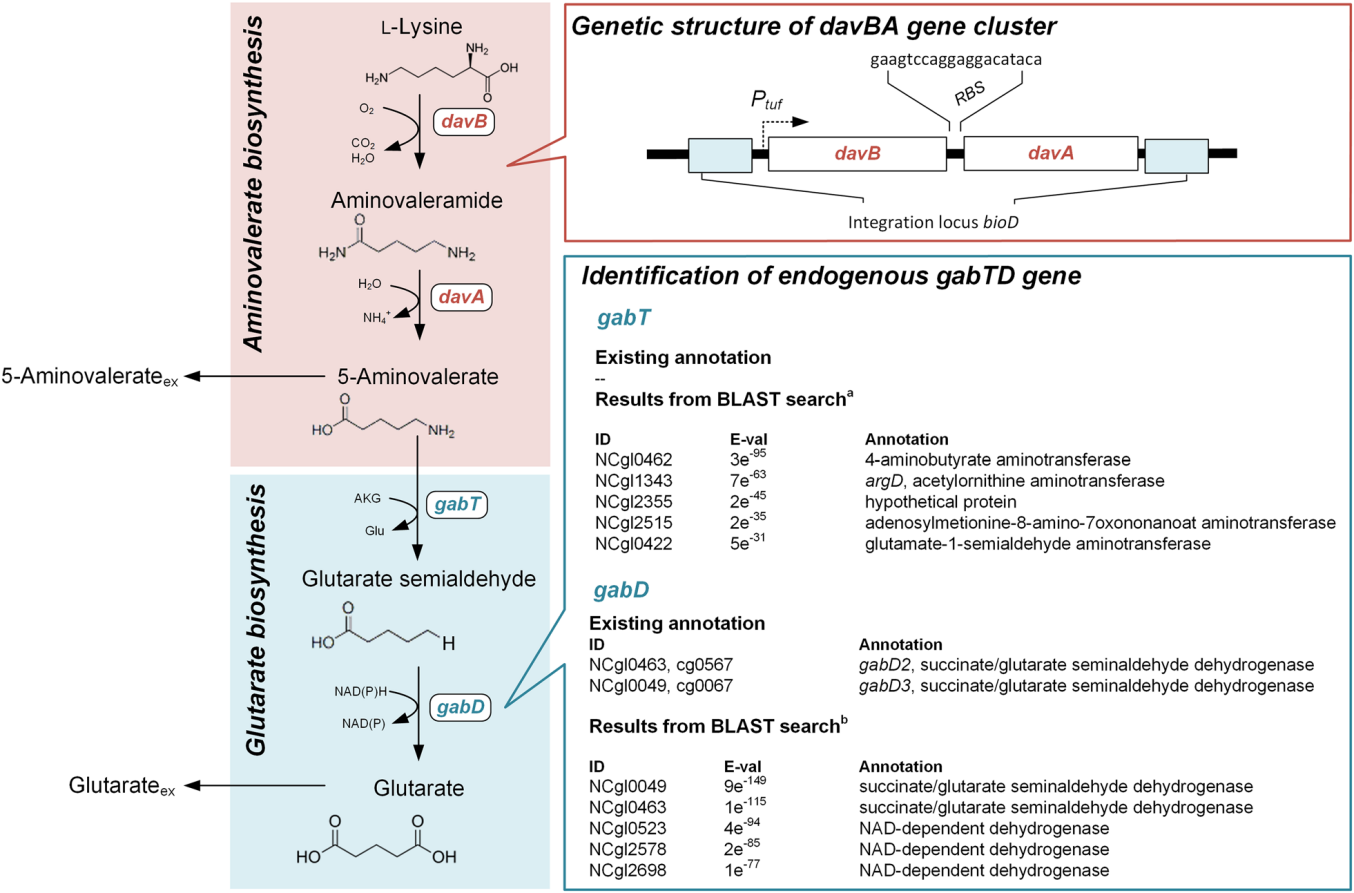
Metabolic pathway design for the production of 5-aminovalerate and glutarate in *Corynebacterium glutamicum* starting from the pathway precursor l-lysine. Heterologous genes (*davBA*) from *Pseudomonas putida* KT2440 were used to reconstruct the 5-aminovalerate pathway comprising lysine monooxygenase (DavB) and 5-aminovaleramidase (DavA). Genes under control of the constitutive *tuf*-promotor were integrated in the *bioD* locus of the genome of *C. glutamicum* LYS-12. Glutarate production from 5-aminovalerate via the activity of 5-aminovalerate transaminase (GabT) and glutarate semialdehyde dehydrogenase (GabD) relied on endogenous reaction with no or promiscuous gene annotation. Potential candidates were identified by BLASTX search using sequence information of the *P. putida* genes *gabT* (*davT*, PP_0213)^*a*^ and *gabD* (*davD*, PP_0214)^*b*^

Today’s petrochemical industry is challenged by the ever-increasing demand for commodity chemicals, polymer materials and related compounds, which is additionally aggravated by the ever-decreasing accessibility of fossil resources as base material [1]. Hence, substantial effort has been made for providing alternative “green” routes to produce platform chemicals through microbial fermentation processes. Compounds of interest comprise organic acids [2] such as succinate [3–5], lactate [6], and itaconic acid [7], diamines including putrescine [8, 9] and cadaverine [10–14] and diols [15–18], all being applicable as building blocks for bio-based plastics. In this regards, also 5-aminovalerate and glutarate are attractive carbon-5 building blocks for the production of nylon from renewable feedstocks [19, 20]. By now, *Escherichia coli* has been in the focus of interest as platform for either de novo biosynthesis [19] of 5-aminovalerate and glutarate or for biotransformation [20–22] thereof from l-lysine. As 5-aminovalerate and glutarate are degradation products of the proteinogenic amino acid l-lysine, we choose the non-pathogenic Gram-positive soil bacterium *Corynebacterium glutamicum*, a well-established industrial l-lysine producer [1], as metabolic chassis for the production of these carbon-5 platform chemicals. Beyond the excellent availability of genetic tools [23], and knowledge of its physiology and large-scale fermentation [24, 25], engineered *C. glutamicum* has only recently been established as centerpiece for the production of l-lysine-derived cadaverine within a pipeline towards the manufacturing of the fully bio-based polyamide PA5.10 [13]. In this work, the metabolic pathway from l-lysine towards 5-aminovalerate was reconstituted by stable genome-based implementation of the *Pseudomonas putida* KT2440 genes *davB*, encoding lysine monooxygenase, and *davA*, encoding 5-aminovaleramide amidase (Fig. 1)—a strategy previously established for *E. coli* [19, 21]. Glutarate production relied on an endogenous pathway (Fig. 1). Though experimentally shown [26], full and unambiguous annotation of the genes coding for the required enzymes 5-aminovalerate transaminase (GabT) and glutarate semialdehyde dehydrogenase (GabD) is missing so far (Fig. 1). We further engineered the basic producer by elimination of by-product formation and focusing the production flux towards 5-aminovalerate. Production was subsequently benchmarked in fed-batch fermentation.

## Results

### Recombinant expression of the *davBA* genes establishes 5-aminovalerate and glutarate production in *C. glutamicum*

**Fig. 2:**
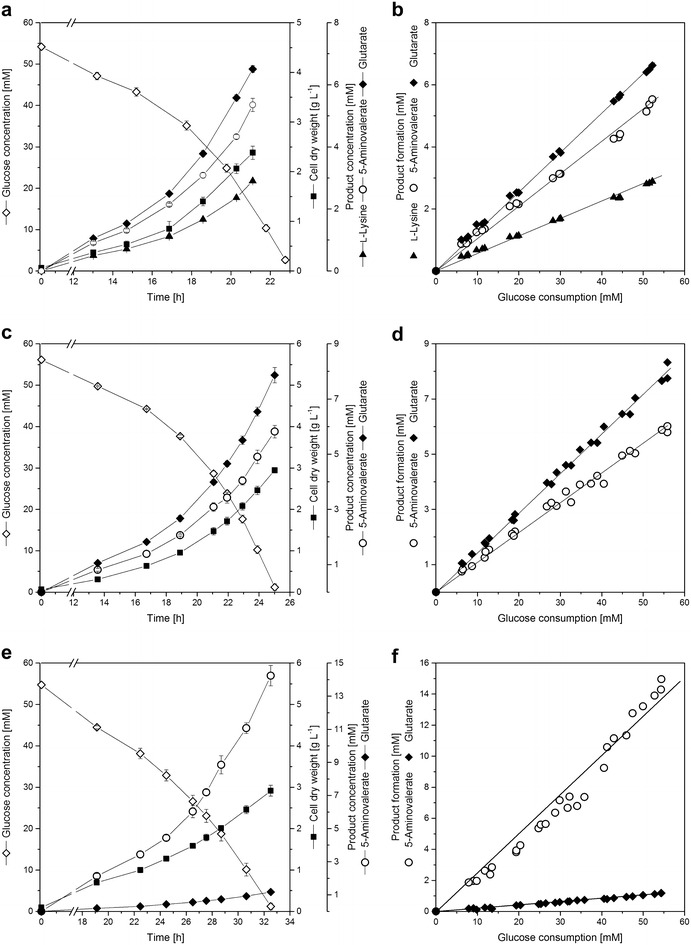
Growth and production characteristics of 5-aminovalerate and glutarate producing *C. glutamicum*. The *C. glutamicum* strains AVA-1 (**a**, **b**), AVA-2 (**c**, **d**) and AVA-3 (**e**, **f**) were cultivated in shake flasks at 30 °C in a chemically defined medium. The cultivation profiles show growth, product formation and glucose consumption over time and represent mean values and corresponding standard deviations from three biological replicates (**a**, **c**, **e**). Yields were determined as slope from the linear correlation of glucose consumption and production formation from three biological replicates (**b**, **d**, **f**)

Upon implementation of the *P. putida* genes *davBA* in the genome of *C. glutamicum* LYS-12, this l-lysine-hyper producing strain was re-programmed for 5-aminovalerate and glutarate production. Genome-based integration of the construct (Fig. 1) was first verified by PCR analysis. Cloning-associated mutations were subsequently excluded by sequencing. Functional expression of lysine monooxygenase (DavB) and 5-aminovaleramidase (DavA) was validated by in vitro activity measurement. The underlying assay allowed parallel investigation of l-lysine consumption, related to DavB activity, and 5-aminovalerate production, related to DavA activity. In crude cell extracts of the recombinant AVA-1 strain both lysine consumption (4.1 ± 0.4 mmol L^−1^ h^−1^) and 5-aminovalerate production (1.8 ± 0.6 mmol L^−1^ h^−1^) was observed. As no conversion took place in the parent strain *C. glutamicum* LYS-12, this could be specifically attributed to the heterologous *davBA* expression. Additional proof for the functional operation of the pathway came from cultivation experiments. During growth of *C. glutamicum* AVA-1 in glucose minimal medium, both 5-aminovalerate and glutarate were produced and secreted into the medium (Fig. 2a). The production was growth-associated. After depletion of glucose, 5-aminovalerate and glutarate accumulation stopped at a titer of 5.4 and 6.5 mM, identifying glutarate as major product in the AVA-1 strain. The pathway precursor l-lysine was also found in the culture supernatant, though it was only secreted to a lower extent of 2.9 mM (Fig. 2a). All products were produced constantly and we could not observe any production shift throughout the cultivation (Fig. 2b), which allowed precise and representative determination of the yields (Table 1). Glutarate was produced most efficiently (123 mmol mol^−1^). In comparison, the 5-aminovalerate yield was 24 % lower and l-lysine secretion only contributed to about 19 % of the total product formation. The observed production pattern indicated that the l-lysine exporter protein LysE obviously competed with the activity of lysine monooxygenase, catalyzing the initial step of the 5-aminovalerate/glutarate pathway. Similarly, 5-aminovalerate export competed with the endogenous transamination reaction (Fig. 1). The preference of the cell to rather form glutarate than 5-aminovalerate indicated that transamination was more efficient than export. However, once secreted, 5-aminovalerate was not channeled back into the glutarate pathway. Indeed, when *C. glutamicum* LYS-12 was grown in the presence of 5-aminovalerate, the concentration of 5-aminovalerate did not decrease over the cultivation time and no glutarate formation was observed.

**Table 1:** Growth and production performance of the *C. glutamicum* strains AVA-1, AVA-2 and AVA-3 during batch cultivation in shake flasks using a mineral salt medium with glucose as sole carbon source

	*C. glutamicum* AVA-1	*C. glutamicum* AVA-2	*C. glutamicum* AVA-3
Rates			
µ [h^−1^]	0.22 ± 0.04	0.20 ± 0.00	0.11 ± 0.00
q_s_ [mmol g^−1^ h^−1^]	4.59 ± 0.70	3.67 ± 0.27	2.16 ± 0.07
q_Ava_ [mmol g^−1^ h^−1^]	0.43 ± 0.06	0.38 ± 0.01	0.59 ± 0.02
q_Glt_ [mmol g^−1^ h^−1^]	0.57 ± 0.07	0.50 ± 0.00	0.05 ± 0.00
q_Lys_ [mmol g^−1^ h^−1^]	0.24 ± 0.04	0.00 ± 0.00	0.00 ± 0.00
Yields			
Y_X/S_ [g mol^−1^]	47.6 ± 3.6	53.9 ± 1.0	51.8 ± 0.5
Y_Ava/S_ [mmol mol^−1^]	97.6 ± 2.0	104.9 ± 1.8	274.9 ± 2.9
Y_Glt/S_ [mmol mol^−1^]	123.4 ± 2.4	140.7 ± 5.6	21.8 ± 1.4
Y_Lys/S_ [mmol mol^−1^]	52.6 ± 0.9	0.0 ± 0.0	0.0 ± 0.0

The data represent mean values and standard deviations from three biological replicates and denote the specific rates for growth (µ), substrate uptake (q_S_), and product formation (q_P_). Additionally, the yield for biomass (Y_X/S_), 5-aminovalerate (Y_Ava/S_), glutarate (Y_Glt/S_), and l-lysine (Y_Lys/S_) are given

### Secretion of the by-production l-lysine is eliminated by the deletion of *lysE*

To account for the undesired secretion of l-lysine in the basic strain AVA-1, the *lysE* gene, encoding for the lysine exporter, was deleted from the genome. For this purpose, a shortened DNA fragment, lacking 575 bp of the coding region of *lysE,* was used to replace the native gene. Correct modification was identified by PCR analysis. Deletion of *lysE* positively affected the production of the desired C5-compounds in the second-generation strain *C. glutamicum* AVA-2. Titer (Fig. 2c) and yield (Table 1) were increased for both glutarate and 5-aminovalerate. l-Lysine excretion was fully eliminated. The excess carbon gained from exporter deletion was mostly used in favor of glutarate production. We also observed an increase in the biomass yield, as compared to the parent *C. glutamicum* AVA-1 strain (Table 1). Obviously, biomass formation appeared as newly arising competitor for the additionally available carbon. The specific growth rate was hardly affected by *lysE* deletion, whereas the specific glucose uptake rate was 30 % lower (Table 1). Overall, this resulted in a decreased specific production rate for both glutarate and 5-aminovalerate, despite the increased yield (Table 1).

### Identification and elimination of 5-aminovalerate transaminase selectively increases the 5-aminovalerate production flux

When aiming at the production of 5-aminovalerate, withdrawal of the product via the glutarate pathway has to be circumvented. The underlying biochemical reactions were, however not fully identified in *C. glutamicum* so far. For *P. putida* it is known, that two enzymes namely 5-aminovalerate transaminase, encoded by *gabT* (*davT*), and glutarate semialdehyde dehydrogenase, encoded by *gabD* (*davD*) are responsible for glutarate formation from 5-aminovalerate [27]. For the glutarate semialdehyde dehydrogenase encoding gene, two potential candidates are annotated in the genome of *C. glutamicum* (Fig. 1). A gene annotation for the 5-aminovalerate transaminase is, however completely missing. To identify potential genes in the genome of *C. glutamicum*, a nucleotide BLASTX search was carried out using the *gabD* and *gabT* sequence of *P. putida* as input. From the obtained set of candidates, hits with best E-value were evaluated in more detail, considering existing annotation and genomic localization (Fig. 1). The most promising candidates were the genes NCgl0462 (*gabT*) and NCgl0463 (*gabD*) as they show high similarity scores to the *P. putida* sequence and exhibit the same genomic organization as the *P. putida* genes as two adjacent open reading frames. For improving 5-aminovalerate production, we selected the *gabT* gene, annotated as 4-aminobutyrate aminotransferase (Fig. 1), as deletion target to immediately cut the glutarate pathway at the level of 5-aminovalerate and to avoid accumulation of the pathway intermediate glutarate semialdehyde. The coding sequence of the gene was fully deleted as validated by PCR analysis, which revealed a 1347 bp-shortened DNA fragment in *C. glutamicum* AVA-3 as compared to the parent strain. The strategy for generating the third-generation strain *C. glutamicum* AVA-3 proved highly beneficial. The product 5-aminovalerate accumulated up to 14 mM (Fig. 2e), which was more than twice as high as compared to the ancestor strains. The mutant exhibited highest yield (275 mmol mol^−1^) and, in particular, highest specific production rate, despite impaired growth and glucose uptake (Table 1). The latter might partly be related to intracellular accumulation of l-lysine, 5-aminovaleramid and 5-aminovalerate as response to the network perturbation at the level of LysE and GabT. Though small, a secretion flux for glutarate remained indicating residual in vivo transamination of 5-aminovalerate. The strong decrease in the glutarate yield (Table 1), however, clearly showed that the major contributing enzyme had been eliminated by NCgl0462 deletion.

### Excellent performance of the 5-aminovalerate-producing *C. glutamicum* strain AVA-3 under fed-batch conditions

**Fig. 3:**
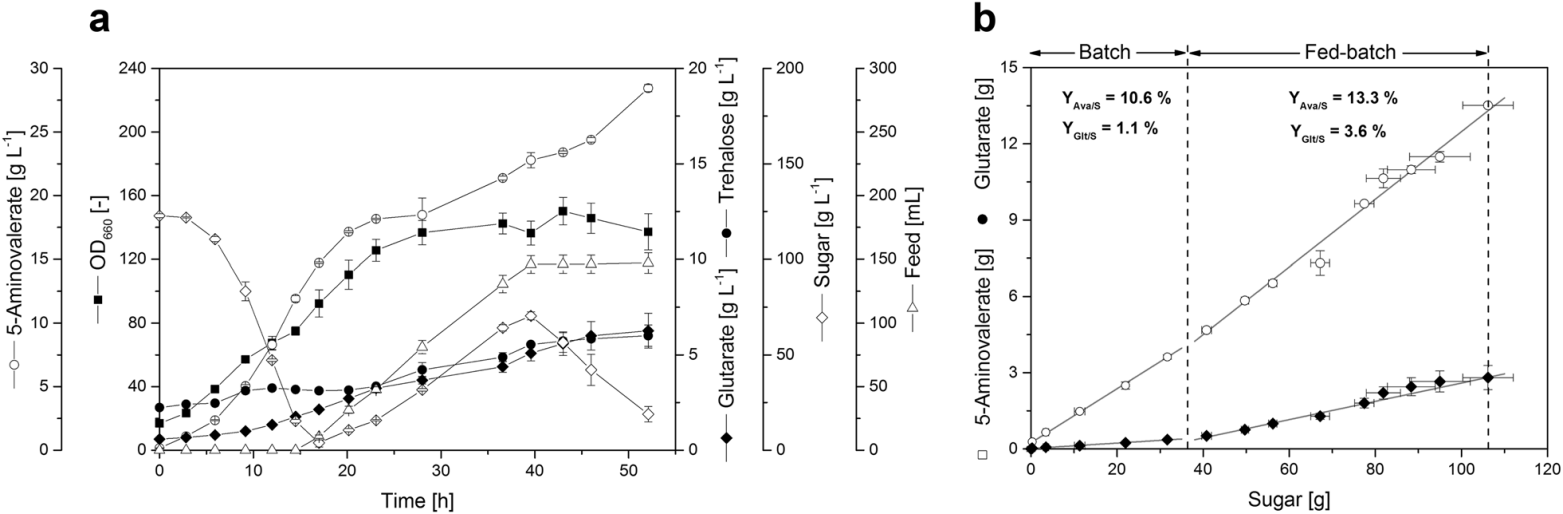
Production performance of the advanced 5-aminovalerate producing strain *C. glutamicum* AVA-3 during fed-batch fermentation. Cultivation profile of strain AVA-3 (**a**), and yields for 5-aminovalerate and glutarate achieved in the different cultivation phases (**b**) are shown. The data represent mean values from two independent fermentation experiments

**Fig. 4:**
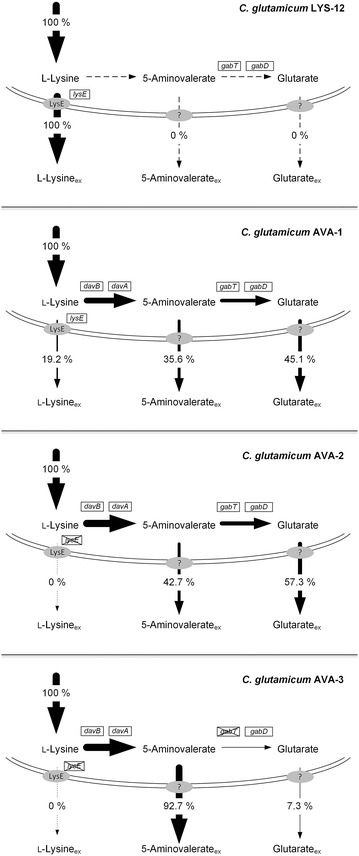
Relative contribution of l-lysine, 5-aminovalerate and glutarate secretion to the overall production flux. The production of l-lysine, 5-aminovalerate and glutarate in *C. glutamicum* LYS-12, AVA-1, AVA-2 and AVA-3 is given as relative flux related the to the overall l-lysine-based production flux, which was set to 100 %

To assess the performance of the designed production strain under conditions more relevant for an industrial process, we benchmarked 5-aminovalerate production by this strain in a fed-batch process on a glucose-molasses medium. In the initial batch phase of the fermentation, we observed exponential growth along with 5-aminovalerate production (Fig. 4a). After a process time of 15 h, the initially supplied sugar (120 g L^−1^) was depleted and the feed phase was started. Feeding pulses were applied to maintain sugar concentration above 20 g L^−1^. The level of 5-aminovalerate continuously increased from 12 g L^−1^ at the end of the batch phase to a final titer of 28 g L^−1^ after 50 h. Glutarate was concomitantly secreted and accumulated up to 7 g L^−1^ (Fig. 3a). Proper determination of the optical density as indicator for biomass concentration was hampered in the feed phase due to cell agglomeration and wall-associated growth. The final biomass concentration of 70 g L^−1^ was hence determined gravimetrically as cell dry mass from the total reactor content at the end of the process. Trehalose was the only other by-product that accumulated in noteworthy amounts (7 g L^−1^). When evaluating the efficiency for 5-aminovalerate production throughout the process phases (Fig. 3b), slightly different yields were obtained for the batch (0.11 g g^−1^) and the feed phase (0.13 g g^−1^). The glutarate yield increased by threefold in the feed phase. The space–time yield for 5-aminovalerate was maximal in the batch phase with a rate up to 0.9 g L^−1^ h^−1^. Over the full process time, production occurred at more than half-maximum rate (0.5 g L^−1^ h^−1^).

## Discussion

In recent years, l-lysine-derived compounds, e.g. cadaverine [13, 28, 29], glutarate [19, 22, 26], 5-aminovalerate [20, 21] and pipecolic acid [30] have emerged as attractive platform building blocks, to establish bio-based production routes for chemicals and materials [31, 32]. This depicts an excellent possibility to employ existing l-lysine overproducers as starting point for re-engineering. *Corynebacterium glutamicum* is the major industrial workhorse for the production of l-lysine [1, 33] and—unlike *P. putida* [27] and *E. coli* [19]—it naturally misses catabolic reactions for degradation of l-lysine. l-lysine overproducing *C. glutamicum* strains are hence ideal metabolic chassis for establishing bio-based fermentation processes for these interesting compounds, as there are no side-reactions competing with the target product formation. In this work, we established a de novo production process for the carbon-5 platform chemicals 5-aminovalerate and glutarate on basis of *C. glutamicum* LYS-12 [34]. These are promising building blocks for bio-based polyamides and polyesters [19], when polymerized with appropriate counterparts such as caprolactame [20] and diamines [13], and beyond serve as platform chemicals [22].

### Expression of *davBA* fills the gap for de novo production of 5-aminovalerate and glutarate in *C. glutamicum*

The strategy of using heterologous expression of the *P. putida* genes *davBA* for re-programming of *C. glutamicum* LYS-12 for 5-aminovalerate and glutarate production proved highly valuable as more than 80 % of the total production flux was drained towards the newly implemented pathway (Fig. 4). Due to the lack of l-lysine catabolic reactions, additional strain modifications, required for *E. coli*-based processes [19, 22], were obsolete. About one-third of the rechanneled carbon was secreted as 5-aminovalerate, whereas the major fraction was further metabolized to glutarate via endogenous reactions (Fig. 4). In *E. coli,* production of 5-aminovalerate and glutarate specifically relied on heterologous expression of the *davAB* cluster (5-aminovalerate) or a combined expression of the *davBA*-*davDT* (*gabDT*) clusters (glutarate) as, in contrast to *C. glutamicum*, endogenous genes for *davDT* are obviously missing [19, 20, 22]. Despite pure production of either of these compounds, *E. coli* reached maximal yields of 71 mmol mol^−1^ for 5-aminovalerate and 68 mmol mol^−1^ for glutarate in a de novo process [19], which is even far below the performance of the basic AVA-1 strain (Table 1). The different production pattern for both species might be related to differences concerning re-uptake of 5-aminovalerate. *E. coli* efficiently converts externally added 5-aminovalerate into glutarate, when the *davBA* and *davDT* (*gabDT*) gene clusters from *P. putida* are concomitantly expressed [19]. The obviously active import system for 5-aminovalerate in *E. coli* can thus explain that there is no mixed production of 5-aminovalerate and glutarate [19, 22]. The constant production characteristics of our novel strains (Fig. 2), however, show that once 5-aminovalerate is secreted, there is no re-import and further conversion to glutarate. In addition, we could not observe any glutarate formation in *C. glutamicum* LYS-12 when 5-aminovalerate was supplied to the medium. This is attributed to the lack of an active 5-aminovalerate uptake system under the tested conditions. Efficient product secretion without re-uptake—as found in this study for *C. glutamicum*—is highly desirable and might even become crucial to establish commercially feasible processes [35–37]. The genome-based engineering approach ensured strain stability as previously proven for manifold genetic manipulations of *C. glutamicum* [38–42]. Overall, production of the target metabolites and the remaining l-lysine production in the AVA-1 strain nicely summed up to the l-lysine production of the parent strain [34]. We took this finding as good indicator that no other by-products arose in noteworthy amounts, as previously shown for cadaverine-producing *C. glutamicum* [10, 11].

### 5-aminovalerate production is limited by the in vivo activity of the heterologous pathway

The secretion of l-lysine in the AVA-1 strain revealed limitations in the in vivo activity of the downstream 5-aminovalerate pathway. The newly implemented pathway operated less efficiently than the cadaverine pathway, previously established in *C. glutamicum*, which fully converted l-lysine into cadaverine by the heterologously implemented lysine decarboxylase [10]. Intracellular l-lysine accumulation in the AVA-1 strain was, however, sufficient to induce expression of the l-lysine exporter [43, 44]. Consequently, the exporter LysE competed with lysine monooxygenase for intracellular l-lysine as substrate, a phenomenon similarly found previously for l-lysine-derived cadaverine production, whereby the cadaverine export protein competed with cytosolic acetyl-transferase reaction that formed N-acetyl-cadaverine [10, 11]. We hence had a closer look at the enzyme activity of lysine monooxygenase and 5-aminovaleramidase. In vitro assays with crude cell extract revealed that the lysine consumption rate, reflecting lysine monooxygenase activity, was two times higher than the production rate of 5-aminovalerate, reflecting 5-aminovaleramidase activity. Expression of both enzymes from the designed operon was obviously not perfectly balanced. For enzymatic production of 5-aminovalerate, however, it was recently shown, that a 1:1 ratio of both enzymes is required for optimal production [21]. On basis of the in vitro activity, the bottleneck of the 5-aminovalerate pathway could on a first glance be assigned to 5-aminovaleramidase. The initial step catalyzed by lysine monooxygenase is, however, massively feedback inhibited by 5-aminovaleramide, 5-aminovalerate, and glutarate at levels of 5 mM [27], likely resulting in a substantially reduced in vivo activity. Moreover, from enzyme activity measurements, carried out in closed tubes, we have additional indication that lysine monooxygenase can become limiting, when oxygen supply is insufficient. In in vitro studies with limited oxygen supply, identical rates for lysine consumption and 5-aminovalerate production were determined (0.56 ± 0.01 mmol L^−1^ h^−1^). We hence cannot clearly attribute the bottleneck specifically to one of the enzymes and/or an imbalanced expression thereof. Elimination of LysE activity was therefore most straightforward to avoid loss of the pathway precursor l-lysine. Lacking any secretion system for l-lysine, the additionally available carbon in the AVA-2 strain was partly used in favor of 5-aminovalerate and glutarate formation, and partly supports biomass production, as similarly found recently for ectoine producing *C. glutamicum* [45]. Despite the higher yields, the specific production rates were not increased as the in vivo activity of the heterologous pathway remained a limiting factor. The rates were actually lower than in the parent strain (Table 1), as the overall fitness (µ, q_S_) of the AVA-2 strain was slightly impaired. Further improvement of the strain performance can thus be expected from higher *davBA* gene expression. Moreover, removal of feedback inhibition of lysine monooxygenase is a highly desirable, though challenging task for future strain optimization.

### The glutarate pathway is the major competitor for 5-aminovalerate production

The obtained production pattern clearly indicated that the endogenous transaminase reaction of the glutarate pathway is dominating over the 5-aminovalerate export. Deletion of the putative transaminase GabT in the AVA-3 strain successfully redirected the production flux to 5-aminovalerate, though glutarate production was not fully eliminated. Obviously, *C. glutamicum* possesses more than one gene and enzyme, responsible for 5-aminovalerate transamination. It is interesting to note that *P. putida*, which can naturally degrade l-lysine via the 5-aminovalerate/glutarate pathway, only possesses a single *gabT* gene variant, the lack of which prevents growth on l-lysine as sole carbon source [46]. Additional deletion of further gene candidates for *gabT* and *gabD* (Fig. 1) appears most straightforward to further diminish glutarate formation in the *C. glutamicum* AVA-3 strain. However, only few is known about their actual function, leaving a certain risk of undesired side effects upon deletion. As alternative strategy, the enhancement of the 5-aminovalerate export could drive a more efficient product secretion. Indeed, transport processes are a major issue of industrial processes and thus often addressed in the production hosts for improving production of e.g. cadaverine [12, 47], amorphadiene [48], l-lysine [49] and l-arginine [50]. In light of the strong feedback inhibition of lysine monooxygenase, the secretion efficiency appears even more relevant. Similar to *E. coli* [19], the transport mechanisms for 5-aminovalerate and associated proteins are not yet known for *C. glutamicum*. The secretory capacity of *C. glutamicum* for non-natural compounds is, however, admirable as demonstrated in recent years for the production of ectoine [45], cadaverine [12], putrescine [8], and γ-amino butyric acid [51]. As shown for cadaverine [12], export is thereby not necessarily linked to a unique and specific transporter. There are likely transporter families with promiscuous secretory activity for structurally related metabolites, which might be of help for identification.

### High-cell density fermentation reveals excellent production performance

To take benefit from high cell density and the generally improved production performance [34, 45], the best 5-aminovalerate producer was investigated during fed-batch operation. *C. glutamicum* AVA-3 produced 28 g L^−1^ of 5-aminovalerate within only 50 h, which is the highest titer and productivity described so far for de novo production of this compound [19, 22, 26]. The maximal space–time yield of about 0.9 g L^−1^ h^−1^ was even within the range of bio-conversion processes directly producing 5-aminovalerate from l-lysine [19–22]. De novo production as described here obviously benefits over such bio-transformation process, as production can be conducted as one-step process directly from cheap raw material, regularly applied for industrial fermentation [24, 25, 52]. In contrast, biotransformation requires successive production steps for the supply of the biocatalyst, e.g. whole cells or purified enzymes, and a subsequent conversion step for product formation. Moreover, it strictly relies on the pathway precursor l-lysine, additionally being produced by fermentation, requiring subsequent purification. In this regard, d*e novo* production is clearly more flexible, related to the broad natural substrate spectrum of *C. glutamicum* and additional engineering of assimilation routes for non-natural carbon sources [53, 54]. Depending on price and availability, alternative renewable feedstocks [29, 40, 55] or carbon-containing waste streams from industry [54, 56] might become more attractive, and could hence be used for production.

## Conclusion

In this work, we established *C. glutamicum* as production platform for the polyamide building blocks 5-aminovalerate and glutarate through genetically stable genome manipulation. Following the strategy of heterologous engineering of the 5-aminovalerate pathway from *P. putida* [19, 22, 26], combined with additional modifications that targeted by-product formation, we generated a strain with the highest de novo production performance concerning titer, yield and productivity. With the remaining issues on the in vivo activity of the *davBA* pathway, 5-aminovalerate export and glutarate production, we can immediately identify future engineering targets towards strain improvement. Further optimization can be expected applying systems metabolic engineering to unravel and circumvent additional and less obvious bottlenecks through iterative rounds of strain analysis, design and genetic engineering. Similar titer, yield and productivity as already achieved for l-lysine [34] and cadaverine [13, 57] production thus appear feasible to leverage performance of 5-aminovalerate to industrial efficiency. Moreover, the AVA-2 strain already builds an ideal basis for generating glutarate overproducing *C. glutamicum* strains.

## Methods

### Microorganisms and plasmids

In the present work, the ATCC 13032-derived l-lysine-overproducing *C. glutamicum* strain LYS-12 [34] was used as host to establish 5-aminovalerate and glutarate production. Reconstruction of the 5-aminovalerate pathway relied on the *davBA* genes, encoding lysine monooxygenase and 5-aminovaleramidase from *P. putida* KT2440. Amplification of transformation vectors on basis of the integrative plasmid pClik int *sacB* [10, 58] was carried out in *E. coli* strains DH5α and NM522 (Invitrogen, Carlsbad, CA, USA) [34]. All strains and plasmids employed in this study are listed in Table 2.

**Table 2:** Description of the *C. glutamicum* strains and plasmids used in the present work for the heterologous production of 5-aminovalerate and glutarate

	Description	Reference
Strain		
* C. glutamicum* LYS-12	Lysine-hyperproducing strain with 12 genome-based modifications	[34]
* C. glutamicum* AVA-1	LYS-12 + genome-based integration of the *P. putida* genes *davB* (PP_0383) and *davA* (PP_0382) encoding lysine monooxygenase and aminovaleramide amidase, into the *bioD* gene locus (NCgl2516), encoding dithiobiotin synthetase	This work
* C. glutamicum* AVA-2	AVA-1 + deletion of *lysE* gene (NCgl1214), encoding the lysine exporter	This work
* C. glutamicum* AVA-3	AVA-2 + deletion of putative *gabT* gene (NCgl0462), encoding 5-aminovalerate transaminase	This work
Plasmids		
pTC	Expression vector for DNA-methyltransferase of *C. glutamicum,* ORI for *E. coli* and tetracycline resistance as selection marker. Used in *E. coli* NM522 to add the *C. glutamicum*-specific DNA-methylation pattern to the integrative transformation vector	[34]
pClik int *sacB*	Integrative transformation vector for *C. glutamicum* with MCS, ORI for *E. coli*, and Kan^R^ and *sacB* as selection markers	[58]
pClik int *sacB tuf*_*p*_-*davBA*	Integrative transformation vector for genome-based implementation of *davBA* genes from *P. putida* KT2440 into the *bioD* locus of *C. glutamicum*	This work
pClik int *sacB ∆lysE*	Integrative transformation vector for deletion of the lysine exporter *lysE*	[12]
pClik int *sacB ∆gabT*	Integrative transformation vector for deletion of the 5-aminovalerate transaminase gene *gabT*	This work

### Molecular design and genetic engineering

For molecular strain, plasmid and primer design, the Clone Manager Professional 9 (Sci-Ed Software, Denver, USA) was used. For sequence similarity search towards identification of *gabT* and *gabD* candidates in the genome of *C. glutamicum*, the corresponding gene sequences from *P. putida* (PP_0214, *gabT, davT* and PP_0213, *gabD, davD*) were BLASTed (BLASTX, http://www.genome.jp/tools/blast/) against the *C. glutamicum* genome, deposited at KEGG (http://www.genome.jp/kegg/). The genetic construct for genome-based integration of the *davBA* gene cluster from *P. putida* KT2440 comprised (i) 500 bp-sized flanking regions as homologous recombination sites for the *bioD* locus (NCgl2516), (ii) a 200 bp-sized DNA fragment of the promotor of the structural *tuf* gene (NCgl0480), and (iii) the biosynthetic genes *davB* (PP_0383) and *davA* (PP_0382), which were separated by a 20-bp sized ribosomal binding site as inter-genic region (Fig. 1). The putative *gabT* gene (NCgl0462) was fully deleted from the genome. All DNA fragments were amplified by PCR (2× Phusion Flash PCR Master Mix, Thermo Scientific, Waltham, MA, USA; peQSTAR, PEQLAB Biotechnology GmbH, Erlangen, Germany) from genomic DNA of *C. glutamicum* ATCC13032 and *P. putida* KT2440, respectively, with sequence specific primers (Table 3). DNA fragment and vector assembly was carried out by the method of Gibson [59]. The reaction mixture contained 157.5 mM Tris–HCl (pH 7.5), 15.75 mM MgCl_2_, 15.75 mM DTT, 42 mg µL^−1^ PEG-800, 0.6 mg µL^−1^ NAD, 25 mU µL^−1^ Phusion™ High-Fidelity DNA Polymerase (Thermo Fisher Scientific, Rochester, New York, USA), 7.5 mU µL^−1^ T5 exonuclease (Epicentre, Madison, USA), 4 U µL^−1^ Taq Ligase (Thermo Fisher Scientific, Rochester, New York, USA), each 0.3 mM dNTPs. Prior to this, the vector was linearized via restriction with *Bam*HI (FastDigest®, Thermo Fisher Scientific, St. Leon-Roth, Germany). Vector amplification in the *E. coli* strains DH5α and NM522, purification of plasmid DNA, and its transformation into *E. coli* and *C. glutamicum* strains were performed as described previously [39]. PCR and sequence analysis (GATC Biotech AG, Konstanz, Germany) were used for plasmid and strain validation. Deletion of the *lysE*-gene encoding the l-lysine exporter of *C. glutamicum* was conducted as described previously [12].

**Table 3:** Description of primers that were used in the present work for genome-based integration of *tuf*-promoter controlled *davBA* genes from *P. putida* in the *bioD* locus (PR_davBA__1–PR_davBA__10) and for deletion of the *gabT* gene (PR_gabT__1–PR_gabT__4) in *C. glutamicum*

No.	Sequence	AT [°C]
PR_davBA__1	CTGCGTTAATTAACAATTGGTAAGCAATGGCCTACAACCAGAC	59
PR_davBA__2	CATTCGCAGGGTAACGGCCAGGTTTATTTCCCTTTAACTGCAGC	57
PR_davBA__3	CAGTTAAAGGGAAATAAACCTGGCCGTTACCCTGCGAATG	62
PR_davBA__4	TGGCGGTTCTTCTTGTTCATTGTATGTCCTCCTGGACTTCGTGGTG	64
PR_davBA__5	GAAGTCCAGGAGGACATACAATGAACAAGAAGAACCGCCACCCCGC	68
PR_davBA__6	GCGCATTGTATGTCCTCCTGGACTTCTCAATCCGCCAGGGCGATCGG	66
PR_davBA__7	CGATCGCCCTGGCGGATTGAGAAGTCCAGGAGGACATACAATGCGCATCGCTCTGTACCAGG	64
PR_davBA__8	CGAAGGCACGGTGTTCACGATCAGCCTTTACGCAGGTGCAG	62
PR_davBA__9	TGCACCTGCGTAAAGGCTGATCGTGAACACCGTGCCTTCG	62
PR_davBA__10	AATCCCGGGTCTAGAGGATCACGCATGAGTGTGCTTGTGGAA	62
PR_gabT__1	CTGCGTTAATTAACAATTGGCGCTGGAGGTGATCGAGATAAATG	59
PR_gapT__2	CTGGGAAGGTCAAAGACACTGGTTCCTCCTGTGAGGTGAGATAC	59
PR_gabT__3	CTCACCTCACAGGAGGAACCAGTGTCTTTGACCTTCCCAG	56
PR_gabT__4	AATCCCGGGTCTAGAGGATCCAAAGACGGAGGCGATGATC	57

### Batch cultivation in shake flasks

*Corynebacterium glutamicum* was grown in baffled shake flasks with 10 % filling volume at 30 °C and 230 rpm on an orbital shaker (Multitron, Infors AG, Bottmingen, Switzerland; 5 cm diameter). The cultivation procedure involved one pre-culture in complex medium (37 g L^−1^ BHI) followed by another pre-cultivation in minimal medium to adapt cells to the medium conditions of the main cultivation [60]. The main cultivation was carried out as triplicate in a chemically defined mineral salt medium with glucose as the carbon source [61]. The medium contained: (A) 500 mL salt solution (1 g NaCl, 55 mg MgCl_2_·7H_2_O and 200 mg CaCl), (B) 100 mL substrate solution (100 g L^−1^ glucose), (C) 100 mL buffer solution (2 M potassium phosphate, pH 7.8), (D) 100 mL solution B (150 g L^−1^ (NH_4_)_2_SO_4_, pH 7.0), (E) 20 mL vitamin solution (25 mg L^−1^ biotin, 50 mg L^−1^ thiamine·HCl and 50 mg L^−1^ pantothenic acid), (F) 10 mL FeSO_4_-solution (2 g L^−1^ FeSO_4_, pH 1.0), (G) 10 mL 100× trace elements [62] and (H) 1 mL DHB-solution (30 mg mL^−1^ of 3,4-dihydroxybenzoic acid in 0.3 M NaOH) adjusted to 1 L with milliQ. Solutions were separately sterilized by autoclaving (A–D) or by filtration (E–H). The different medium compounds were combined at room temperature freshly before use.

### Fed-batch cultivation in stirred tank bioreactors

The production performance of the optimized 5-aminovalerate-producer *C. glutamicum* AVA-3 was evaluated in a fed-batch process. This was carried out in a complex molasses-based medium, containing per liter: 72.4 g beet molasses, 35 mL corn steep liquor, 40 g (NH_4_)_2_SO_4_, 99 g glucose·H_2_O, 250 µL H_3_PO_4_ (85 %), 11 mg FeSO_4_·7H_2_O, 10 mg citrate, 9 mg biotin, 17.5 mg thiamine·HCl, 60 mg pantothenic acid Ca-salt, 18 mg nicotinamid, 6 mg riboflavin, 6 mg FAD, 250 mg KH_2_PO_4_, 100 mg MgSO_4_. The initial batch was started in a volume of 300 mL in a 1000 mL bioreactor (SR0700ODLS, DASGIP AG, Jülich, Germany) and inoculated with cells as previously described [13]. Dosing of the feed (650 g L^−1^ glucose·H_2_O, 162.5 g L^−1^ beet molasses, 40 g L^−1^ (NH_4_)_2_SO_4_, 6 mg L^−1^ riboflavin, 6 mg L^−1^ FAD) was initiated when the sugar concentration dropped below 20 g L^−1^ and adjusted to maintain glucose concentration constant above 20 g L^−1^. The cultivation temperature was kept constant at 30 °C via the CWD4 bioblock (DASGIP AG, Jülich, Germany). The pH and the pO_2_ level were monitored online with a pH electrode (Mettler Toledo, Giessen, Germany) and a pO_2_ electrode (Hamilton, Höchst, Germany). The pH was kept constant at 6.9 ± 0.1 by automated addition of 25 % NH_4_OH (MP8 pump system, Eppendorf, Hamburg, Germany). The dissolved oxygen level was maintained at saturation above 30 % by variation of the stirrer speed and the aeration rate. Data acquisition and process operations were controlled by the DAGIP control software (DASGIP AG, Jülich, Germany).

### Substrate and product quantification

Amino acids and 5-aminovalerate were quantified by HPLC, using α-aminobutyric acid as internal standard [63] with a modified gradient (0 min: 70 % eluent A; 14 min: 56 % eluent A; 14.5 min: 0 % eluent A; 16.5 min: 0 % eluent A; 17 min: 70 % eluent A; 19 min: 70 % eluent A). Samples from fed-batch fermentation were additionally diluted with MilliQ water prior to dilution with the internal standard to account for the detector limit (<1.5 mM). Quantification of glucose and organic acids was carried out by isocratic HPLC (Agilent 1260 Infinity Series, Waldbronn, Germany) comprising a degasser (G4225A), an autosampler (G1329B) including thermostat (G1330B), an isocratic pump (G1310B), a column oven (G1316C), and a refraction index (RI) detector (G1362A). Separation was conducted on an Aminex HPX-87H column (300 × 7.8 mm; Bio-Rad, München, Germany) at 55 °C. As mobile phase 3.5 mM H_2_SO_4_ was used at a flow rate of 0.8 mL min^−1^. The injection volume was 20 µL. For sugar analysis of supernatant samples from fed-batch fermentation, a Metacarb 87C column (300 × 7.8 mm; Agilent, Darmstadt, Germany) with Metacarb 87C guard column (50 × 7.8 mm; Agilent, Darmstadt, Germany) and additional Micro-Guard De-Ashing Refill Cartridges (Bio-Rad, München, Germany) was used at 85 °C with demineralized water as mobile phase at a flow rate of 0.4 mL min^−1^. If appropriate, samples were diluted prior to analysis. Refraction index was used for detection. Concentration of cell dry mass (CDM) was calculated from the optical density using a correlation factor of CDM [g/L] = 0.32 × OD_660_. This was determined as described previously [61] for the here used UV-1600PC spectrophotometer (VWR, Hannover, Germany).

### Determination of enzyme activity

Crude cell extracts were prepared from exponentially growing cells by mechanical cell disruption. Cultivation and harvest procedure was carried out as previously described [29]. Aliquots of 1 mL cell suspension were then transferred into FastPrep®-24 vials (MP Biomedicals, Illkirch-Graffenstaden, France) containing silica beads (Ø 0.1 mm) and cell disruption was carried out in 2 × 30 s cycles at 5000 rpm (Precellys®-24, PeqLab, Hannover, Germany) including 1 min cooling pauses on ice. Removal of cell debris and protein quantification was performed as previously described [61]. The protocol for subsequent quantification of the conversion rate of l-lysine into 5-aminovalerate, comprising the lumped activity of lysine monooxygenase and 5-aminovaleramide amidase, was based on previous protocols [19, 21]. The final reaction mixture of 5 mL contained 100 mM phosphate buffer (pH 7.5), 15 mM l-lysine, and 50 µL mL^−1^ of crude cell extract (0.5 mg mL^−1^ of protein). Incubation was carried out at 30 °C and 230 rpm on an orbital shaker (Multitron, Infors AG, Bottmingen, Switzerland) in 50 mL baffled shake flasks. Samples were regularly taken and thermally inactivated (5 min, 100 °C, Thermomixer F1.5, Eppendorf, Hamburg, Germany). l-lysine consumption and 5-aminovalerate formation were quantified by HPLC as given above. Negative controls were conducted without the addition of crude cell extract and l-lysine, respectively. Moreover, the crude cell extract of the parent strain *C. glutamicum* LYS-12 was analyzed to account for potential non-DavBA associated enzymatic reactions.

## Abbreviations

Ava: 5-aminovalerate
AKG: α-ketoglutarate
*bioD*: encoding gene of dithiobiotin synthetase
*davA*: encoding gene of aminovaleramide amidase
*davB*: encoding gene of lysine monooxygenase
*gapT*: encoding gene of 5-aminovalerate transaminase
*gabD*: encoding gene of glutarate semialdehyde dehydrogenase
Glt: glutarate
Glu: glutamate
Lys: l-lysine
LysE, *lysE*: lysine exporter and corresponding gene
MCS: multiple cloning site
ORI: origin of replication
PCR: polymerase chain reaction
*sacB*: encoding gene of levansucrase from *Bacillus subtilis*
*tuf*: encoding gene of elongation factor tu
